# Predicting Individual Action Switching in Covert and Continuous Interactive Tasks Using the Fluid Events Model

**DOI:** 10.3389/fpsyg.2016.00023

**Published:** 2016-01-27

**Authors:** Gabriel A. Radvansky, Sidney K. D’Mello, Robert G. Abbott, Robert E. Bixler

**Affiliations:** ^1^Department of Psychology, University of Notre DameNotre Dame, IN, USA; ^2^Department of Computer Science and Engineering, University of Notre DameNotre Dame, IN, USA; ^3^Sandia National LaboratoriesAlbuquerque, NM, USA

**Keywords:** event cognition, action change, behavior prediction, language comprehension, video games, mental updating

## Abstract

The Fluid Events Model is aimed at predicting changes in the actions people take on a moment-by-moment basis. In contrast with other research on action selection, this work does not investigate why some course of action was selected, but rather the likelihood of discontinuing the current course of action and selecting another in the near future. This is done using both task-based and experience-based factors. Prior work evaluated this model in the context of trial-by-trial, independent, interactive events, such as choosing how to copy a figure of a line drawing. In this paper, we extend this model to more covert event experiences, such as reading narratives, as well as to continuous interactive events, such as playing a video game. To this end, the model was applied to existing data sets of reading time and event segmentation for written and picture stories. It was also applied to existing data sets of performance in a strategy board game, an aerial combat game, and a first person shooter game in which a participant’s current state was dependent on prior events. The results revealed that the model predicted behavior changes well, taking into account both the theoretically defined structure of the described events, as well as a person’s prior experience. Thus, theories of event cognition can benefit from efforts that take into account not only how events in the world are structured, but also how people experience those events.

## Fluid Events Model: Event Comprehension

When people do a task, they do not always progress in the same way throughout the task. Instead, they alter their actions as a function of the nature and demands of the task, and as a result of their own experiences. For example, during a football game, a coach may call certain plays in part of because of the position of the teams on the field, the amount of time on the clock, the score of the game, the weather, and other factors related to the circumstances being faced. However, the play call selection is also influenced by the coach’s recent experiences in terms of the success or failure of various plays that have been called up to that point. We contend that both the structure of the environment and a person’s own prior experiences both influence whether a decision is made to continue with the current action or change to a new one. This is a broad-based issue that can pervade a wide range of human activity, and the ability to model such shifts would be useful to researchers trying to predict behavior along a task that persists over long periods of time.

We have recently developed the Fluid Events Model of event cognition to explain changes in people’s actions within interactive events (Radvansky et al., 2015). The driving force behind the model is the idea that behavioral selection is guided by both the structures of the events people encounter, as well as their prior experiences. As outlined by Radvansky et al. (2015), there are three general types of events in which performance can be assessed. These are (a) the more covert processing of events, as with narrative comprehension, (b) active interaction with a series of discrete trials, and (c) active interaction with continuous events. The Radvansky et al. (2015) study assessed the Fluid Events Model in the context of event types of the second type. The aim of the current paper is to present an assessment of this model using events of the first and third type.

The application to the more covert processing of events is important because the majority of studies in event cognition involve people processing events that they are watching or reading about, with little in the way of overt behavior. The application to active interaction with continuous events is important because the interactive tasks that have been assessed, like much of the work in cognitive psychology, have involved people performing actions across a series of trials in which the starting state of each trial is largely independent of what happened on the trial before. In the real world, independent trials are the exception rather than the rule. Instead, our actions often change the state of the world, altering the circumstances for subsequent choices. The current project extended the Fluid Events Model to these more dynamic interactive events. Thus, the overall aim of the current study was to explore whether the principles embodied in the model can be successfully applied to a wide variety of event processing tasks.

### Covert Event Processing

As already mentioned, the previously reported work with the Fluid Events Model addressed the ability to predict discrete actions on independent trials. For example, people in one task drew copies of figures presented to them, and performance was assessed in terms of how people drew those figures (i.e., the starting points, and the ordering of the components) on a trial-by-trial basis. However, in other common situations, such as narrative events (e.g., reading a text), people receive information with the only observable action that is available might be reading time, eye tracking^1^, or any other measure that reveals the intake of information. We consider these more covert event processing situations first.

Because comprehension is more covert, individuals make relatively few overt actions that reveal how they are processing the events as they unfold. One overt assessment of comprehension that can be capitalized on is reading time. While reading, the time taken to process a sentence before moving on may reflect various aspects of cognitive processing. Of particular concern here is any increase in reading time as a result of factors that are not strongly tied to the text itself (e.g., syllable count) but that occur as a result of a change in the underlying event structure, such as a character moving from one location to another, or the introduction of a new character. Numerous studies (e.g., Zwaan et al., 1995; Zwaan and Radvansky, 1998; Zwaan et al., 1998; Radvansky et al., 2001; Rinck and Weber, 2003; Radvansky and Copeland, 2010; McNerney et al., 2011) demonstrate that such changes reflect the processing of underlying event structure. Thus, the action here is the updating of one’s mental model understanding, and the event being assessed is the processing of the text. In other words, in the context of the Fluid Events Model, we interpret the switch in the underlying event structure described by a text as a change in the environment the person is processing, and a change in reading time as reflecting a switch from one event model to another. This is a change in the action on the part of the comprehender because they are transitioning from processing one narrative event to another.

Event segmentation judgments are another method to assess event comprehension (Newtson, 1973; Newtson and Engquist, 1976; Newtson et al., 1977, Speer et al., 2007; Zacks and Swallow, 2007; Zacks et al., 2007; Magliano and Zacks, 2011), albeit in a more covert manner. This task presents people with a narrative in which they are to indicate those points at which they perceive that a new event has begun. The instructions provided are intentionally vague for how people should segment the events, yet different individuals make remarkably consistent judgments. The majority of studies examine segmentation of narratives in film, but printed narratives have also been studied (e.g., Magliano et al., 2012). This segmentation task is like the reading time task in that the action being assessed is the shift from one mental representation to another. Within our Fluid Events Model, indicating a new segment constitutes a cognitive action on the part of the comprehender. The difference between reading time measures and event segmentation tasks is that the first reflects more unconscious and implicit mental processes, whereas the second reflects more conscious and explicit mental processes.

### Continuous Interactive Events

This paper also describes the application of the Fluid Events Model to continuous interactive events, which are characterized by interdependent sequences of actions. Our prior studies focused exclusively on independent trials. Here we also consider on-going continuous events where each action may constrain or enable subsequent actions. For example, when one is playing a game, the positon one finds oneself in is a function of those moves that have been made previously, and the success of those prior moves. Again, these are the types of events that people are more likely to have to deal with in their everyday experiences, so understanding how people process these experiences is important to understand.

We defined continuous interactive tasks within the context of computer games and derived Fluid Events Model predictions of in-game action changes. The actions defining performance were different for each scenario. While there are wide array of possible actions that can be taken, we selected those actions, in each case, that can at least potentially result in a change in the state of the unfolding events, such as inflicting damage on one’s opponent. In general, the Fluid Events Model predicts when a participant will select a new strategy during the course of game play as a function of the both the current state of the game and the participant’s prior experience. That is, whether a person elects to engage in a different action is a consequence of both the current state of the game, at that moment, as well as the experiences the person has had during game play, particularly involving their more recent experiences, and the consequences of their actions.

## The Fluid Events Model

**FIGURE 1:**
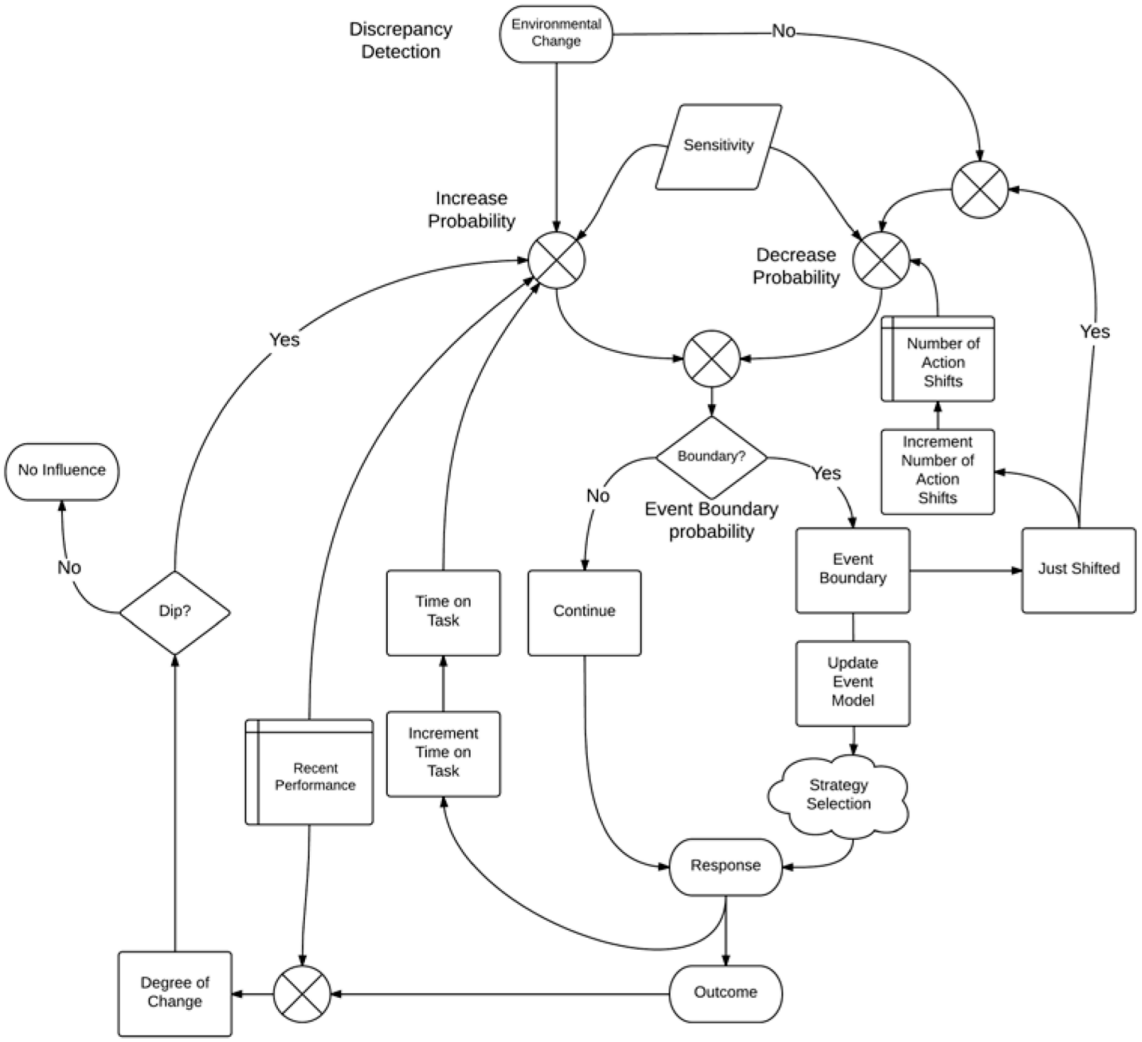
**Overview of the Fluid Events Model**.

The Fluid Events Model estimates the probability that an individual will change from one mode of action to another within the context of a single on-going task. In the case of covert event processing, this corresponds to event segmentation. For continuous interactive events, the model predicts a shift from one action to another. Again, it is important to keep in mind that the model does not predict *which* action will be taken, but instead predicts a *shift* from some action to another *within the same task*. The Fluid Events Model operates on a total of seven factors, which can be divided into two categories: event-structure, and experience-based (these two clearly would interact to some degree, depending on the circumstances). The nature of the task determines the amount of influence from each factor. An overview of the model is provided in **Figure 1** and the equations and fixed values of factors used by the model are provided in **Table 1**.

**Table 1:** Fluid Event Model Factors.

**Fluid Event Models the probability of an action shift as the sum of factors:**
**Bad Shift**
Bad Shift doubles Performance Dip if the previous trial was a strategy shift.
**Flexibility**= F_-1_.1(H_-1_ - P(H)_-1_)
Flexibility is the base rate (i.e., prior probability) of an action shift for the person. It is initialized to 0 and updated according to the learning rate (0.1) and error term for the previous prediction, _H_-1_-P(H)_-1__ where _H_-1__ is 1 if strategy shifted on the previous trial and 0 otherwise, and _P(H)_-1__ is the output of the Fluid Events Model, i.e., the probability of shift on the previous moment.
**Number of Action Shifts**= 1.001^-s^ - 1
S is the total number of action shifts by this person (across all conditions).
**Performance Dip**= 1 - 1.1^-c-1^
Where the decrease C = S_min_-S_t-1_/S_max_-S_min_, where _S_t-1__ is the performance measure on the previous trial and _S_min__, _S_max__ are the minimum and maximum scores from the 10 preceding _S_t-1__ .
**Just Shifted** = -.01R^-R-1^
WhereR is the number of trials since the previous action shift.
**Task Shift**
This factor is task-dependent and reflects recent changes in the situation that might prompt a change in action. Task Shift was between 0 and 0.1 in 38% of trials, between 0.4 and 0.5 in 10% of trials, and greater than 0.6 in 1% of trials.
**Time on Task**= 1 - 1.001^-A-1^
*A* is the total number of trials (across all conditions) done by the person.

### Event-Structure Factors

There are two factors in the Fluid Events Model that involve event-structure: (1) *Environmental Change* reflects recent event structure changes; and (2) *Time on Task*.

*Environmental Change* must be defined for each task to quantify recent alterations to the task or operating environment. For example, changing road conditions may prompt a driver to alter speed, following distance, and aggressiveness of braking. Alternatively, a football coach may be less likely to call pass plays in a game if the rain fall becomes increasingly intense. Environmental Change influences the probability of an action shift externally, with that probability increasing in proportion to the size of the change. Environmental Change is zero when there is no change from the prior event.

*Time on Task* is an event-structure factor that assumes that the longer a person has been doing a particular action, the greater the probability that a change to a new one will occur. As a person grows familiar with a task, they may experiment with other actions. This is similar to the idea in event cognition research that as people progress through an event, the serial position of new information can influence processing (Stine-Morrow et al., 1996). Time on Task is an event-structure factor because it plays a role in the model that is separate from the individual’s actions and their consequences. Time on Task is defined either as the number of events that have occurred before the current time, for those tasks that have discrete trails, or as a certain amount of time that has passed, for those events that ae more continuous. This component is always involved in the calculation of the probability of an action switch. Time on Task is operationalized in the Fluid Events Model as a slow-moving power function that uses the event number as its input (see **Table 1**). Power functions accurately model many psychological functions (e.g., Stevens and Galanter, 1957; Wixted and Ebbesen, 1991), motivating their use here.

### Experience-Based Factors

Along with Event-Structure factors, the Fluid Events Model incorporates Experience-Based factors that are grounded in each person’s unique on-going experience. These experience-based factors are: (1) *Just Shifted*: whether a person recently changed actions; (2) *Number of Action Shifts*: how often had the person already changed actions; (3) *Performance Dip***:** whether a decline in performance has recently been experienced; (4) *Bad Shift*: whether a performance dip was a consequence of an action shift; and (5) *Flexibility***:** the person’s current bias to try different actions.

*Just Shifted* reflects whether the person has selected a new action recently. If so, they are less likely to switch again in the near future, as the new action is given a chance to be effective in improving performance. For example, after a coach has put a new player into a football game there will be a bias against taking that player out immediately thereafter to see if he has an impact on the progress of the game. *Just Shifted* only applies in the absence of an environmental change that drives a change in action. Because this part of the model only involves recent performance, it is only active when a change has been made recently; otherwise it has no bearing on the predictions. This principle is implemented in the Fluid Events Model as a fast-moving power function that quickly diminishes over time (see **Table 1**). Because this is a decrease in the likelihood of an action shift, this is why the formula in **Table 1** is negative. Also, note that this element would not be involve in the prediction of an action shift at the beginning of a task because there is no prior action with which to compare the current action to determine that there has been an action shift.

*Number of Action Shifts* is the number of action changes that have occurred so far during the task. As this value grows, the probability of further changes diminishes. For example, as the number of different types of plays that have been tried by a football coach increases, all things being equal, there will be a decreased willingness to try untested strategies. Number of Action Shifts is a power function; with small values having a greater influence than large ones (see **Table 1**). That is, the fewer actions that have been tried, the greater the probability that a new action shift will occur.

*Performance Dip* increases the probability of an action change after a decline in performance, which may prompt a change in tactics (Reder and Schunn, 1999). For example, if a car is losing speed climbing a hill, the driver might try to accelerate. Alternatively, if a player is doing poorly in a football game, a coach would be more likely to pull him, or if attempts to blitz the quarterback have failed and have resulted in the other team making big advances (or even scoring), then the coach would be more likely to call different plays. For the Fluid Events Model, the greater the decline relative to recent performance, the greater the probability of an action change occurring. Performance dip is a power function, based on the extent to which performance falls below the minimum level attained in the recent past (i.e., the probability of an action change will not be affected if a small dip in performance is within the range of recent performance). The influence of *Performance Dip* grows with larger deviations from the recent minimum (see **Table 1**). Because recent performance drives this part of the model, it only is involved when a dip is outside of the recent range. As an additional component, if a performance dip occurred just after an action shift, then the model will trigger the inclusion of a *Bad Shift* component. The effect of this is to double the effect of any consequence of a Performance Dip. The reasoning behind this is that a performance dip after an action shift might indicate that the new action is inappropriate for the task, and the person would be even less likely to continue it.

Finally, there are individual degrees of *Flexibility* in the probability that an action switch will occur. A person’s momentary trend toward changing increases or decreases the action shift probability. For example, if a coach has been shifting strategies often throughout a game, they would be predicted to be more likely to continue to do so as the game progresses. In the Fluid Events Model, this is done in a simple way using a running average of the difference between the predicted probability that an action change will occur, and whether it actually happened. This factor has a continuous influence.

The Fluid Events Model makes predictions by summing the influences that are operating at that time. Which components enter into this summation is a function of the task and recent experience. Importantly, it is typical that several components do *not* enter into the calculation at a given moment. There is a large existing literature on action selection, but that is not our focus. In an ongoing task, the default is to continue the previous action, so action selection occurs only after the decision to make a change. The Fluid Events Model is specifically aimed at estimating the probability of an action change, and is agnostic as to the decision processes involved in selecting which action to take.

### Example Fluid Event Model Process

To illustrate how the Fluid Event Model operates we have an example from our Red Ace aerial combat task (detailed below). In this task, people play a videogame in which they fly a WW I fighter plane, with the task of destroying enemy planes and ground targets. The environmental change for this task involved the terrain the player was over, and other entities within a zone of interaction (e.g., other planes, ground target, and anti-aircraft guns). The three actions that can be recorded are (1) flying only, (2) firing guns, or (3) dropping bombs.

The values and influence of various components on each of 20 time bins is shown in **Table 2**. For the purposes of exposition, we trimmed the values to be no longer than four decimal places. The score value is an index of performance based on how well the player is doing with 0.5 as a starting point, reduced values indicating problems (e.g., the player being hit by enemy fire), and increased values indicating successes (e.g., destroying targets). Also included in this table is an indication of the number of components contributing to the prediction, the model’s prediction (from 0 to 1), whether a person changed actions or not (1 or 0, respectively), and the action evident on that trial (1 = flying, 2 = shooting, and 3 = dropping bombs).

**Table 2:** Example of the derivation of prediction value for the Fluid Events Model using data from a given attempt of a person on the Red Ace WW I aerial combat video game.

Time bin	Score	Prediction	Switch	Action	Environmental change	Time on task	Just shifted	No. of actions shifts	Performance dip	Bad shift	Flexibility	No. Components
1	0.5	0	0	1	0.05	0.0020	0	0	0	0	0.1600	3
2	0.6	0.2630	1	3	0.1	0.0030	0	0	0	0	0.1600	3
3	0.5	0.2267	1	1	0	0.0040	-0.01	-0.001	0	0	0.2337	4
4	0.5	0.6131	1	2	0.1	0.0050	0	-0.002	0.0995	0.0995	0.3110	6
5	0.5	0.3427	0	2	0	0.0060	-0.01	-0.003	0	0	0.3497	4
6	0.6	0.4694	1	1	0.15	0.0070	0	-0.003	0	0	0.3155	4
7	0.5	0.4225	0	1	0.05	0.0080	0	-0.004	0	0	0.3685	4
8	0.5	0.4295	1	2	0	0.0090	-0.0013	-0.004	0.0995	0	0.3263	5
9	0.5	0.3782	1	1	0	0.0099	-0.01	-0.005	0	0	0.3833	4
10	0.4	0.5004	0	1	0.05	0.0109	0	-0.006	0	0	0.4455	4
11	0.5	0.4995	0	1	0	0.0119	-0.0013	-0.006	0.0995	0	0.3954	5
12	0.5	0.3523	0	1	0	0.0129	-0.0001	-0.006	0	0	0.3455	4
13	0.4	0.3682	0	1	0.05	0.0139	0	-0.006	0	0	0.3103	4
14	0.4	0.4818	1	2	0.1	0.0149	0	-0.006	0.0995	0	0.2734	5
15	0.5	0.3241	0	2	0	0.0159	-0.01	-0.007	0	0	0.3252	4
16	0.5	0.3013	1	1	0	0.0168	-0.0013	-0.007	0	0	0.2928	4
17	0.5	0.3625	0	1	0	0.0178	-0.01	-0.008	0	0	0.3627	4
18	0.5	0.3359	0	1	0	0.0188	-0.0013	-0.008	0	0	0.3264	4
19	0.3	0.4546	1	2	0.15	0.0198	0	-0.008	0	0	0.2928	4
20	0.2	0.7253	1	1	0.15	0.0208	0	-0.009	0.1081	0.1081	0.3474	6

As can be seen, Time on Task and Flexibility have values from the beginning. For time bin 1, the prediction is forced to be zero because at that point there is no information on which to base a prediction. For that first time bin, there were three components involved. These were Environmental Change, because the task is starting, Time on Task, and Flexibility. Flexibility is not 0 here because prior to this particular data segment the person had already done a number of flights, so there was some basis for estimating the degree of flexibility of action change. For time bin 2, there again were three factors involved (Time on Task and Flexibility, which are always involved), and Environmental Change again. Flexibility is not changed because at the beginning of any task there is no basis on which to estimate any change in flexibility. The sum of these three probabilities is the basis for the prediction (0.1 + 0.0030 +0.1600 = 0.2630). Although this value is low, the person did switch actions from flying to dropping bombs. For time bin 3, there were four factors involved. These were Time on Task and Flexibility again, along with Just Shifted and Number of Actions Shifts. The third factor was involved because the person changed actions from the prior time bin. The fourth is involved because a person changed actions. The prediction is the sum of the values for these four components (0.0040 - 0.01 - 0.001 + 0.2337 = 0.2267). This time bin had an action changed because the person was now only flying. For time bin 4, there are five model components involved. In addition to Time on Task and Flexibility, the person encountered Environmental Change (e.g., two enemy planes), and an increased number of Number of Actions Shifts. Moreover, during the previous time bin, the performance score went down (likely from getting hit), so there was now a contribution of Performance Dip. Moreover, because this performance dip occurred just after an action shift, there is also an influence of Bad Shift. Together the probability of a shift in action is the sum of these factors (0.1 + 0.005 - 0.002 + 0.0995 + 0.0995 = 0.6131), which also happened to result in another shift in action to firing machine guns. Note that, although people had switched actions on the pervious trial, there was an Environmental Change, so Just Shifted is not included in the calculation. From here, one can work out how the model performs for the other time bins in this example.

## The Data Sets

To assess how well the Fluid Events Model predicts action shifts, it was fit to several data sets. Some of these involved the segmentation of traditional narrative events involving written narratives and picture stories. The other data sets involved the interaction of a person with one of several video games. Some basic information about each data set is provided in **Table 3**.

**Table 3:** Point-biserial correlations comparing the Fluid Events Model prediction with actual action shifts, as well as a randomized reordering of those shifts.

Model	No. of subjects	No. of trials	Actual shifts	Randomized shifts
Reading time 1 (young)	48	13728	0.227	0.018
Reading time 1 (old)	48	13728	0.227	-0.098
Reading time 2 (young)	72	13728	0.190	-0.002
Reading time 2 (old)	72	13728	0.187	-0.003
Text story segmentation (young)	28	4480	0.251	0.011
Text story segmentation (old)	28	4480	0.312	-0.002
Picture story segmentation (young)	29	4640	0.244	-0.022
Picture story segmentation (old)	28	4480	0.314	0.009
Risk	17	441	0.345	-0.047
Red ace	15	18664	0.195	0.001
Quake	15	20400	0.317	-0.001

### Narrative Events

#### Reading Time Data

The first narrative comprehension data set is from a study by Radvansky et al. (2001) in which people read narratives. In this study, people read texts that were presented one clause at a time, and the reading times were collected for each one. In Experiment 1, there were four texts that described historical events, such as the tulip craze in the 17th century Netherlands, whereas in Experiment 2 these texts were modified to describe more modern circumstances, such as the beanie baby craze of 20th century America, and presented as fictional narratives. We used reading time as a dependent measure of the cognitive actions taken by people as they comprehended these texts. Theoretically, we view a change in reading time as reflecting a change in the situation model in the mind of the reader (e.g., Zwaan et al., 1995). That is, the reader has interpreted the text as referring to a new event that is different from the prior one, requiring that a new event model needs to be created to mentally capture these events. This creation of a new event model takes time, so an increase in reading time at these points in interpreted as when a person stops using one event model and begins using a new one. Although the behavioral action of the reader is quite minimal, this is taking “action” in the broad sense in the sense that using one event model is different from using another one.

More specifically, for the data itself, we used reading time in milliseconds per syllable, as this largely corrects for the overall length of a text (Lorch and Myers, 1990). We then classified reading times as (a) normal reading time, (b) slow reading time, or (c) fast reading time. Normal reading time was within one standard deviation of a person’s mean reading time, whereas slow and fast reading times exceeded one standard deviation above or below the mean, respectively. Note that reading time speed ups are occasionally observed. For example, in a study by McNerney et al. (2011), participants read an entire novel, and reading times were recorded for each sentence, and then analyzed using the Event Indexing Model. In some cases, readers were reading faster at event boundaries. These seemed to be cases where the event shift was strongly predicted by the information that preceded it (e.g., the earlier text would have foreshadowed that a character would go to a particular location, and then the character went there). Note also that both these experiments involved a comparison of younger and older adults. As such, the data from these two age groups are treated separately here.

For the environment-based factors of the Fluid Events Model we used aspects of the text as coded for the Radvansky et al. (2001) study. Specifically, for the Environmental Change factor, the text had been coded for both the number of new arguments (nouns) as well as shifts in the narrative events as defined and coded in the original study using the Event Indexing Model (e.g., Zwaan and Radvansky, 1998). For simplicity, we coded each change along any of these dimensions as a 0.1 increase in the probability of an action change in the model. So, for example, two new arguments in the text accompanied by a change along the time dimension is coded as 0.3, whereas a change along four event dimensions but without new arguments is coded as 0.4. For the Time on Task component, we use the series position of each text clause. That is, we assumed that the overall task began with the reading of each text, and that the reading of each subsequent clause on a computer screen corresponded to the next time period.

The experience-based factors reflect the person’s own experience with the task. The Just Shifted factor was triggered by each change in the person’s rate of reading (low, medium, or high). The Number of Action Shifts was the number of times that there was a reading time change. Flexibility reflected the frequency of reading rate shifts. The Performance Dip factor was not used because performance (e.g., reading comprehension) was not measured.

#### Boy, Dog, Frog Stories Segmentation

In addition to using reading time to indicate when a person has moved from using one event model to another, explicit event segmentation is another index of a switch from one event model to another as a reader explicitly designates each event boundary. This is an adaptation of the Newtson (1973) procedure in which a person watches an unfolding event and indicates when an event boundary has occurred. While both the reading time and explicit segmentation measures can be viewed as reflecting the segmentation of the flow of action into separate events, the reading time data is more likely to reflect unconscious, implicit processes, whereas explicit segmentation is more likely to reflect conscious, explicit processes. Despite this, the concern here is the idea that the probability that such segmentation would occur as a function of both the more objective structure of the event, as often suggested by most theories of event segmentation, as well as the more subjective experience of the flow of events.

We used the event segmentation data from a study reported by Magliano et al. (2012). In this study, people read a series of six stories by Mayer (1967, 1969, 1971, 1973, 1974, 1975) about a Boy, a Dog, and a Frog. In one experiment, the original picture-versions of the stories were used, with no captions. The task was to indicate when a change in the depicted events occurred. In a second experiment, we used text-based versions of stories that were derived from the pictures. The segmentation judgments constituted actions within the Fluid Events Model. Thus, for these studies, there are two possible actions – either indicating an event segment change, or not. As in Radvansky et al. (2001), this study involved a comparison of younger and older adults, so these data will be treated separately.

Further following Radvansky et al. (2001), narrative event shifts in the Boy/Dog/Frog text were coded. This included changes in spatial location, temporal framework, the introduction of a new character, whether there was a strong affective reaction by one of the characters, changes in the goals of a character, and the start or end of an action sequence. The presence of such changes was used as an index of Environmental Change for the Fluid Events Model. As with the reading time data, each of these changes was simply designated as resulting in a 0.1 increase in the probability of a change, with multiple changes being summed together. For the Time on Task component, we used each picture/text sentence as a measure of unfolding time.

The experience-based factors were defined as follows: Just Shifted was triggered by each event segmentation judgment. Number of Action Shifts was defined as the number of times a participant made an event segmentation judgment. Flexibility was defined as the frequency with which the person made event segmentation judgments within the preceding time window. Finally, because we did not attempt to quantify the performance of the reader, the Performance Dip component was not active in the model.

### Interactive Events

This section covers the application of the Fluid Events Model to continuous interactive tasks, in which the set of actions and available information at each moment depends on the actions leading up to them. This is unlike the reading tasks in the previous section (processing of scripted events) and previous studies of discrete, independent trials typical of laboratory work (e.g., binary prediction). Here, we present three interactive tasks: a computerized version of the strategy board game *Risk*, a World War I flight simulator/combat game, *Master of the Skies: The Red Ace*, and a first person shooter game, *Quake II*. Note that while there was likely some variation in whether participants had played these games before, and this expertise could have influenced performance here, this information was not collected at the time the data were gathered. The aim of the present work was to assess whether the Fluid Events Model could be extended to such work. After this is established, future work could look at individual differences, such as different levels of expertise.

#### Risk

In this task, we used a computerized version of the classic board game Risk, called Risk II (MicroProse, 2000). In this game, players use their armies to capture territory on a simplified world map. In addition to the participant, there were five computer players for each game that the participant was playing against. Each participant played 1 h per day for 3 days. In our analysis, each game was treated as a new condition.

We used a variant, *Capital Risk*, in which each player has one country designated as a capital. Instead of capturing every country, the aim of *Capital Risk* is to capture the other players’ capitals while defending one’s own. In this version of the game, a player can use any country they occupy to attack multiple enemy countries in a single turn, specifying for each attack the number of armies to use. Thus, this task occupies a middle ground between work on discrete tasks that we have reported previously, and the other games described in the following section. Specifically, actions in *Capital Risk* are discrete, but the state of each turn is dependent on the actions taken in the prior turns.

Performance on this task was indexed as a function of the number of countries and armies a player possessed at the end of each turn. Actions were divided into three general “strategies.” These were defined as a function of the number of attacks designated by the participant on a game turn relative to the number of un-countered attacks on the participant by other computer players. Play was categorized as *Aggressive*, *Balanced*, or *Timid* if the number of attacks launched by the player was greater than, equal to, or less than the number of un-countered attacks on the player during that turn, respectively.

The environment-based factors were defined as follows: for Environmental Change, we used the change in the number of enemy players adjacent on the board from the previous turn. For Time on Task, we advanced time by one for each turn.

The experience-based factors were defined as follows: Just Shifted was triggered by a change in the action taken by the player, on the scale of Aggressive/Balanced/Timid. The Number of Action Shifts was the number of such changes since the start of the game. Performance Dip became a factor when player’s performance was lower than on the previous trial. Finally, flexibility was based on the presence or absence of an action change relative to the prediction for that turn.

#### Red Ace

Some aspects of performance in the Red Ace video game (Small Rockets, 2000) have been reported previously by Copeland et al. (2006). In this task, using a joystick, players flew World War I allied warplanes over a variety of terrains against both ground and air targets, some of which shot back. Players were able to control their altitude, speed, and whether they fired their machine guns, dropped bombs, or fired rockets. Performance was assessed in terms of successfully hitting targets, which could be enemy planes, enemy anti-aircraft guns, bridges, or buildings. Moreover, performance also included avoiding getting hit by enemy gunfire, either from enemy planes or from anti-aircraft guns. Players were able to determine which direction they should fly based on sketch map in the upper left hand corner. Actions within this context were assessed in terms of whether a pilot was firing guns, dropping bombs, firing rockets, or simply flying. Each player flew several missions, starting the next mission after the completion of each mission goal.

For the environment-based factors we considered changes in the game events conveyed along the dimensions outlined by the Event Indexing Model (e.g., Zwaan and Radvansky, 1998). These included changes in spatial location (the landscape one was flying over, such as whether it was farmland, a town, a lake, and so on), Entities (friendly and enemy planes, anti-aircraft guns, and ground targets), and Goals (when a designated target was destroyed and a new one had to be selected). For the Time on Task component, we divided the game play into 5 s time windows, starting with when the player began a new mission, and defined a unit of time as one window each. Time advanced with each new time window.

For the experience-based factors, for Just Shifted we used any case where there was a change in the player actions (shooting, bombing/firing rockets, or neither). The Number of Action Shifts was the number of times different actions were taken in subsequent time windows. Performance Dip was involved when there was a lowering of the players’ health level as a result of being hit by enemy gunfire from either enemy planes or ground-based anti-aircraft guns. Flexibility was based on the presence or absence of an action change relative to what was predicted.

#### Quake

Some aspects of performance in the Quake video game (Id Software, 1997) were previously reported by Magliano et al. (2014). In this task, players move through a virtual environment trying to shoot other players while avoiding being shot by them. Movement was controlled using a joystick, and the firing of weapons was done by pressing a trigger on that joystick. This game play involved navigating a virtual environment made of several buildings and rooms within those buildings. Enemy characters would appear at locations predetermined by the game. Performance was assessed in terms of hitting targets (enemy combatants) while avoiding being hit by attacks from those enemies. Actions in his task were assessed in terms of whether the player fired a weapon at an enemy within a time window, or took some other action, such as running away. People played multiple levels of the game, moving from one level to the next when the player reached a target location. At that point the game would automatically move the players on to the next scenario.

As with the Red Ace video game, for the environment-based factors, we considered changes in the game events conveyed along the dimensions outlined by the Event Indexing Model (e.g., Zwaan and Radvansky, 1998), including changes in spatial location [such as when a player moved from one room to another, entities (primarily enemy targets), and goals (when a target was destroyed and a new one needed to be selected]. Again, event shifts were defined and coded for in the original study, and these was used as Environmental Changes for the Fluid Events Model used here. For the Time on Task component, at the start of each level of game play, we divided the game play into 5 s bins, and treated a new bin as an increase in time.

In terms of the experience-based factors, for Just Shifted we based this on where there was a change in the action taken by the player. For the Number of Action Shifts, this was the number of times a person altered their behavior. Performance Dip was involved when there was a lowering of the players’ health level as a result of being hit by enemy gunfire. Flexibility was based on the presence or absence of an action change relative to what was predicted.

## Quick Hits

First, we take a quick look at how well the model does at predicting the probability of an actual shift. For each of the seven tasks we computed point-biserial correlations, as shown in **Table 3**, comparing the predicted probability of an action shift with whether such a change actually occurred. As can be seen, these were positive and moderately large. Moreover, as also shown in the table, we randomized the action change data and compared this with the predicted values and found no relationship, showing that the model is making meaningful predictions and not simply imposing structure on any data set.

**FIGURE 2:**
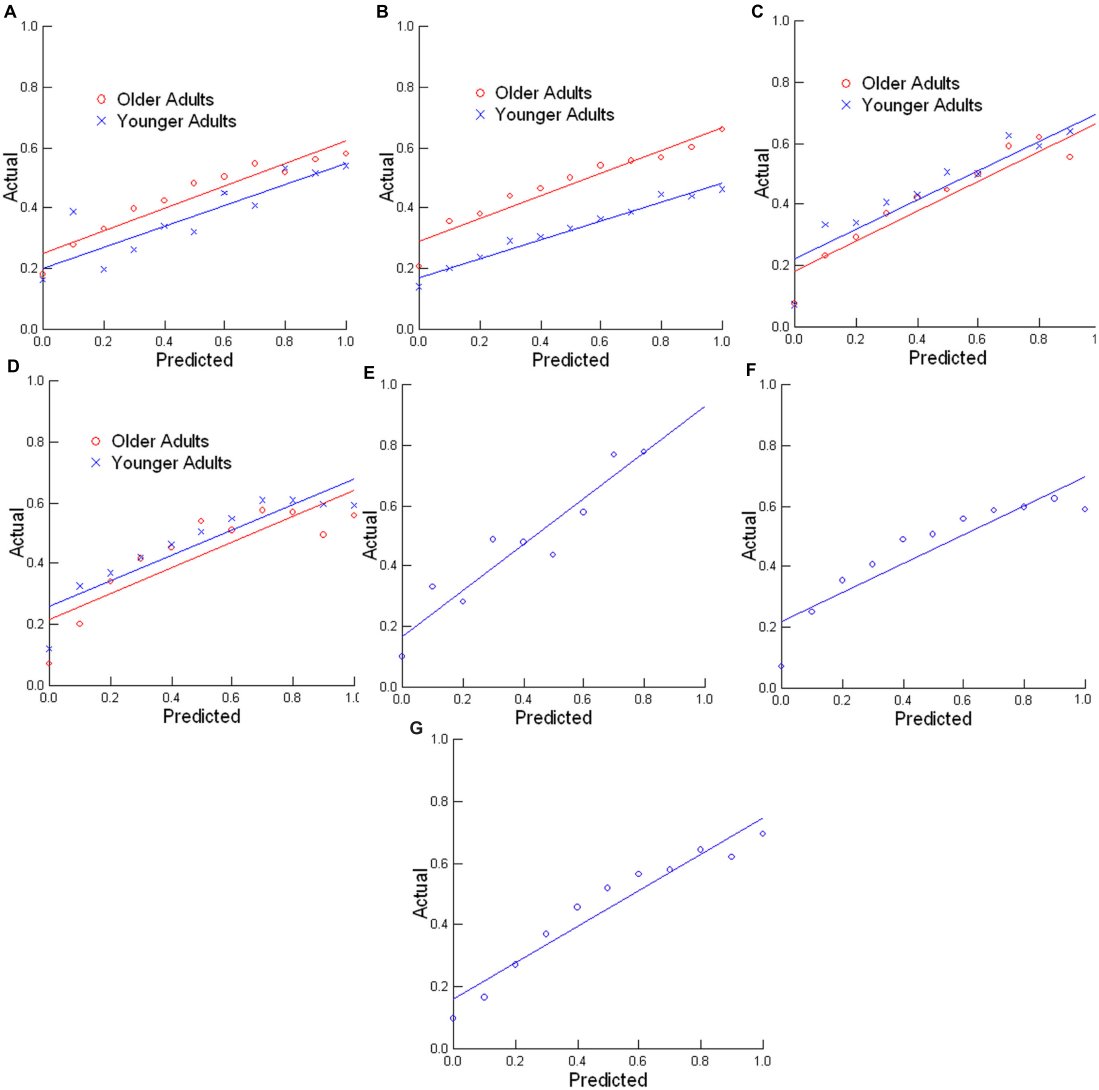
**Comparison of predicted and actual performance when data is distributed in bins for the (A) Reading Time 1, (B) Reading Time 2, (C) Written Story Segmentation, (D) Picture Story Segmentation, (E) Risk, (F) Red Ace, and (G) Quake tasks**. Predicted refers to the predicted probability of an action shift, whereas actual refers to the actual rate of actions shifts.

Furthermore, we also placed the trials into 11 bins based on the predictions generated by the model (i.e., 0, 0.1, 0.2, …, 1.0), and took the average actual shift rate. For example, for the average shift rate, if the predicted values from the model were from 0.35 to 0.44999…, these were put in the 0.4 bin. Then we considered the rate at which people actually changed actions on those trials. Ideally, complete correspondence between these two scores would be found. For example, for the 0.4 model prediction probability bin, there would be action shifts on 40 percent of the trials. The probability graphs for the Reading Time 1, Reading Time 2, Written Story Segmentation, Picture Story Segmentation, Risk, Red Ace, and Quake tasks are shown in **Figures 2A–G**, respectively, along with best fitting linear functions. As can be seen in these graphs, the model made consistent predictions when the data are assessed in this way. Thus, the model does well, at least in its consistency across a wide range of data from different tasks, and without adjustments to any of its parameters.

## Model Fits

The objective of the Fluid Events Model is to capture factors that predict shifts in action for a given task. The internal parameters of the model, which are the constants in **Table 1**, did not change across the data sets, and are the same as used by Radvansky et al. (2015). The model yielded predictions, which were the sum of individual factors, of the likelihood that a person will switch actions from moment to moment. To convert this from a percentage likelihood of a change occurring into a binary yes (1) or no (0) decision we could have selected an *a priori* threshold, which is equivalent to using a step function for decision making. In place of this simplistic approach, a logistic function was used to predict the probability of action switching (1 = switch, 0 = no switch) at the trial level from the factors used by the model. The logistic function converts the model’s values into a predicted switching probability.

Five models were constructed to assess the behavior of the Fluid Events Model. The first was a Null model that included the current trial number as a predictor. The question is whether the Fluid Events Model predicts the action switches net of the Null model. The second was the Fluid Events Sum model, in which case the predictor was the sum of the individual factors; this is the primary model of interest. The third model was the Fluid Events Individual model that used all of the factors as individual predictors rather than using their sum as in the Fluid Events Sum model. This was done to assess if there were added benefits to using the logistic regression framework to assign weights to the factors. The last two models individually considered the event-structure and experience-based factors separately to compare their predictive power. Specifically, the Fluid Events Event-structure model used the *Environmental Change*, and *Time on Task* factors, while Model 5 or the Fluid Events Experience model, used the *Just Shifted*, *Number of Action Shifts*, *Performance Dip*, and *Flexibility* factors. This set of models allows us to make comparisons of the Fluid Events models to the null model, the Event-structure and Experience models, the combined Event-structure and Experience models to the individual models, and summative factor model to individual factor model.

A leave-one-participant-out cross-validation strategy was done to determine whether the models would generalize to new participants. Assuming a data set with N participants, a model built from the combined data of N-1 participants (training data) was used to generate predictions on the data from the “held-out” participant (testing data). This was repeated N times so that each person was “held-out” once. The predictions generated on all of the “held-out” test participants were then analyzed to compute performance metrics. Thus, the training and testing data were always independent at the participant-level, which ensures generalizability to new people.

## Model Accuracy

**FIGURE 3:**
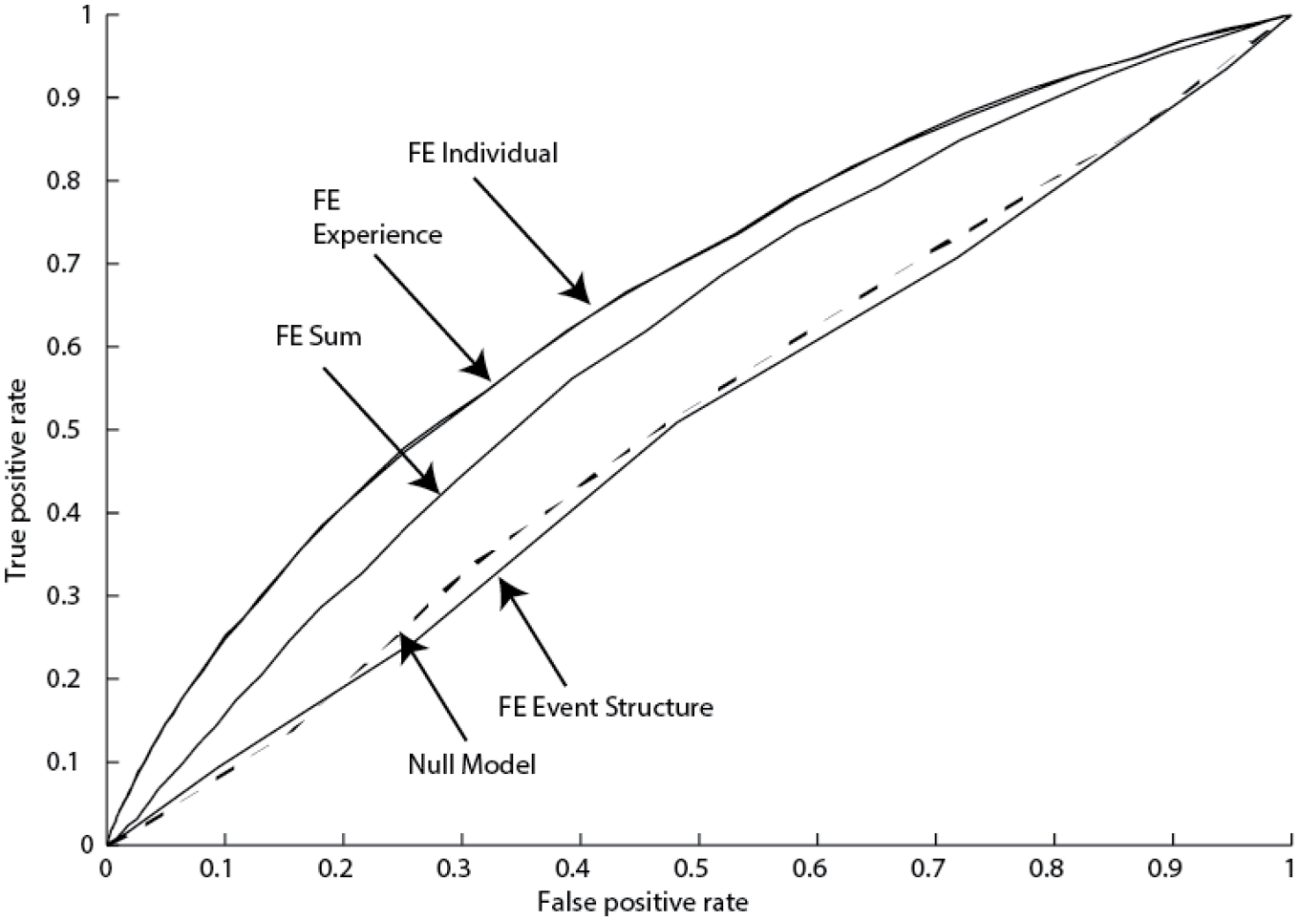
**Receiver operating characteristic (ROC) curves for Reading Time 2 (Younger Adults) data set (FE, fluid events)**.

Receiver operating characteristic (ROC) curves were constructed for the various models. These curves were constructed from two trial-level data streams – the probability of a switch from a logistic regression model (ranges from 0 to 1) and whether there was an actual switch or not (1 or 0). The AUC (area under the ROC curve) metric was used to evaluate model fit and is presented in **Table 4**, with one example worked out in **Figure 3**. An AUC of 1 indicates perfect accuracy, while an AUC of 0.5 indicates chance performance.

**Table 4:** AUC (area under receiver operating characteristics curve) for the various models.

Model	Null	Sum	Individual	Event structure	Experience
Reading time 1 (young)	0.36	0.62	0.69	0.47	0.69
Reading time 1 (old)	0.51	0.61	0.67	0.51	0.67
Reading time 2 (young)	0.51	0.61	0.66	0.50	0.66
Reading time 2 (old)	0.52	0.58	0.62	0.52	0.62
Picture story segmentation (young)	0.57	0.63	0.65	0.57	0.63
Picture story segmentation (old)	0.46	0.68	0.69	0.51	0.68
Written story segmentation (young)	0.52	0.63	0.64	0.59	0.59
Written story segmentation (old)	0.52	0.68	0.69	0.56	0.66
Risk	0.56	0.68	0.70	0.55	0.71
Red Ace	0.51	0.59	0.61	0.54	0.59
Quake	0.50	0.68	0.73	0.66	0.62
**Mean**	0.50	0.64	0.67	0.54	0.65

*Chance performance would obtain an AUC of 0.5*.

Three major conclusions can be drawn. First, the Fluid Events Sum model outperformed the null model (Trial No.) across all of the data sets. On average, this model yielded a 27% performance improvement compared to the null model. Second, optimizing the weights of the individual factors (i.e., the Fluid Events Individual model) resulted in only a small 5% improvement over the basic model that sums all factors. Third, the Fluid Events Experience factors were the major driver of model performance compared to the Event-structure factors. In fact, models built solely from the three Event-structure factors rarely outperformed the null models, with the Quake data being a clear exception. Thus, much of what drives a person’s action switches is their prior experience, not aspects of the task itself. Finally, combining the Event-Structure and Experience factors resulted in very small improvements (3%) over the Experience factors alone because the Experience factors were the major drivers of performance.

We took a closer look at the Fluid Events Individual models, as they involve all of the factors and resulted in the best overall performance. Classification tables were derived by comparing the model predictions to the observed action shifts. The logistic regression model provided a probability of an action switch and these were converted into binary yes/no switch decisions. A threshold of 0.5 was used to discriminate switches (probability ≥ 0.5) from non-switches (probability < 0.5). From these tables, we computed precision (proportion of correctly classified switches) and recall (proportion of total switches correctly identified as switches). **Table 5** shows these scores along with the actual switch rate (prior probability of a switch). We note relatively consistent and moderate precision and recall scores (mean of 0.63 and 0.64, respectively) despite the overall low switch rate (mean of 0.39). Note also, that the *r*^2^ values for the Risk task is lower than the others. This may reflect a more coarse-granularity of this task relative to the others because there were a larger number of individual actions that went into the determination of the overall strategy a person took on a game turn, and fewer trials overall. Still, the model does well even with this smaller data set.

**Table 5:** Performance metrics for the Fluid Events Individual Model.

Model	Switch rate	Precision	Recall	Pearson *r*
Reading time 1 (young)	0.29	0.62	0.63	0.94
Reading time 1 (old)	0.41	0.62	0.63	0.94
Reading time 2 (young)	0.25	0.70	0.76	0.82
Reading time 2 (old)	0.48	0.59	0.59	0.98
Picture story segmentation (young)	0.47	0.60	0.60	0.96
Picture story segmentation (old)	0.40	0.62	0.63	0.91
Written story segmentation (young)	0.44	0.59	0.60	0.98
Written story segmentation (old)	0.37	0.63	0.65	0.90
Risk	0.30	0.64	0.68	0.58
Red ace	0.49	0.57	0.57	0.93
Quake	0.42	0.66	0.66	0.97
**Mean**	0.39	0.63	0.64	0.89

In addition to trial-by-trial action switch prediction, it is also informative to obtain an overall rate of switching for a given person. This is an easier task because we are interested in how many switches occurred rather than when switches occurred. Correlations between the Fluid Events Individual model’s switch rates and overall switch rates (see **Table 3**), computed at the participant-level, were remarkably high (>0.90) for all but two of the data sets.

## General Discussion

This paper presents the further application of the Fluid Events Model (Radvansky et al., 2015), which predicts when a person is likely to abandon one course of action when dealing with unfolding events and pursue another. Again, the focus of this model is not on predicting *which* behavior will be selected, but on the *probability* of an action shift *within the same task*. This would be useful to researchers seeking to predict when the course of action taken by a person, across a wide range of activities, alters from one strategy to another. The Fluid Events Model accomplishes this by integrating numerous factors about the task itself, as well as recent experiences of the individual, that are known or thought to affect the probability of a person changing the actions they take within an on-going course-of-events.

Our previous exposition of the model (Radvansky et al., 2015) found high accuracy in predicting action changes in laboratory tasks in which each trial was largely independent of the others, and in which the tasks were presented in a trial-by-trial manner. Across those tasks, it was found that the probability of a person switching from one action to another, while being influenced in some ways by the demands of the task itself, was more strongly influenced by the experiences of the individual in the task. The aim of the current study was to go beyond the more laboratory-based tasks of this previous work and apply the model to a wide variety of more real-world comprehension- and action- based tasks that go beyond the characteristics of interaction, trial independence, and discrete trials.

The Fluid Events Model was applied to a number of data sets. After fitting the model to the data, it was found to be reasonably accurate in predicting action shifts, with performance being largely driven by the experience-based factors. Instead, the model was able to robustly predict a range of tasks that can influence whether a person will switch strategies. This involvement of a complex set of factors is what would be expected in real-world situations.

### Task Interactivity

In terms of task interactivity, many comprehension tasks require only minimal overt interaction on the part of the comprehender. During reading, people need only pass their eyes over the words, and perhaps hold a paper or book in their hands and turn pages. Despite this lack of overt action, there is still considerable mental activity occurring. It is changes in these mental actions that we aimed to predict in the current study, with a focus on the event model level of cognition (Radvansky and Zacks, 2011, 2014; Radvansky, 2012).

In our reading time and event segmentation tasks, which both looked at the shift from one narrative event to the next, comprehension required minimal interaction with the primary task. We defined action as processing one event model or switching to a new one, as evidence by an increase in reading time (e.g., Zwaan et al., 1995) or the explicit indication of an event boundary (e.g., Zacks et al., 2009; Magliano et al., 2012). What we found was that, while such changes were influenced by structural aspects of the materials, there was also a strong influence of a person’s own prior event model processing. This was true across written and picture-based narratives. Thus, the model was successfully applied to more covert actions. This reflects the idea that there is a great deal of fluidity and ambiguity into how narratives may be comprehended even in terms of how a person breaks them up into the events that are identified.

### Trial Interdependence

In terms of trial independence, many laboratory tasks require performance on a series of largely independent trials in which the participant can start anew on each trial, and performance on the prior trials does not necessarily need to extend to performance on the current trial (leaving carry-over effects, such as proactive interference, aside for the moment, but only focusing on task demands). Participants, at some level, know that how they did on the prior trials often has no bearing on what they are to do on the current trial, other than the basic task remains the same.

In our video game tasks, although aspects may reset at the beginning of a new game, participants knew that what they were doing at any moment of a game was dependent on the actions they took previously. This was true across a range of different games, including both turn-by-turn and continuous action games. This can also been seen to be true, to some extent, for the narrative comprehension tasks in terms of the idea that one’s understanding of what was currently happening in the story was dependent on knowing, to some degree, what had happened previously in the story. Even with tasks that did not have more traditional trial independence, the model accurately predicted action switches, again with a stronger influence of a person’s prior experience. This reflects the idea that people are drawing on their own prior experiences with a task, and how it is unfolding over time, to determine whether they will continue to approach it in the same manner as they had in the past, or to change their behavior in some way to deal with the demands presented to them at the moment.

### Continuous Tasks

In terms of the use of discrete trials, many comprehension tasks require continuous processing of a stream of information, written or visual. Much of the information is not neatly divided into well-defined trials. How well can we define and track a person’s actions, and decisions to change the actions taken, within such tasks?

Except for the game of Risk, this study examined continuous tasks. Even for the written and picture-based narratives, although there were individual sentences and pictures, the story was relatively continuous. What we found across all of these continuous tasks is that we can reasonably define points along which people are more or less likely to change the actions they are taking to do the task. This suggests that, even in a continuous processing environment, people are constantly evaluating how they are approaching and doing a task, and altering their actions accordingly based on both the demands of the task itself, as well as their on-going experience with the task.

## Conclusion

The current study was a further assessment of the Fluid Events Model of event cognition which predicts the likelihood of an action change within the context of the same task. Like prior work (Radvansky et al., 2015), we found that the model predicts when people are likely to alter their actions within a task at a rate well above of chance. Thus, we now have a means of predicting, on a moment to moment basis, the likelihood that a person will change what they are doing. Of particular interest is that, in most of our tasks, the probability of changing actions is determined primarily by the person’s prior experience with the task, rather than the structure of the task. This fits into a larger aim of understanding how people comprehend the world around them, and how they interact with it.

## Author Contributions

GR developed the theory, programmed the cognitive model, designed the data collection studies, and analyzed the data. SD developed the theory and analyzed the data. RA developed the theory. RB developed the theory and analyzed the data.

## Conflict of Interest Statement

The authors declare that the research was conducted in the absence of any commercial or financial relationships that could be construed as a potential conflict of interest.

## Footnotes

1 Eye tracking is generally more informative than simple reading times because it can provide information about individual word processing, regressions, and so on.

## References

[B1] Copeland D. E. Magliano J. P. Radvansky G. A. (2006). “Situation models in comprehension, memory, and augmented cognition,” in *Human Cognitive Models in System Design*, eds Bernard M. Forsythe J. C. Goldsmith T. (Malwah, NJ: Erlbaum), 37–66.

[B2] Lorch R. F. Myers J. L. (1990). Regression analyses of repeated measures data in cognitive research. *J. Exp. Psychol. Learn. Mem. Cogn.* 16 149–157. 2136750 10.1037//0278-7393.16.1.149

[B3] Magliano J. Kopp K. McNerney M. W. Radvansky G. A. Zacks J. M. (2012). Aging and perceived event structure as a function of modality. *Aging Neuropsychol. Cogn.* 19 264–282. 10.1080/13825585.2011.633159 PMC3347870 22182344

[B4] Magliano J. P. Radvansky G. A. Forsythe J. C. Copeland D. E. (2014). Event segmentation during first-person continuous events. *J. Cogn. Psychol.* 26 649–661. 10.1080/20445911.2014.930042

[B5] Magliano J. P. Zacks J. M. (2011). The impact of continuity editing in narrative film on event segmentation. *Cogn. Sci.* 35 1489–1517. 10.1111/j.1551-6709.2011.01202.x 21972849 PMC3208769

[B6] Mayer M. (1967). *A Boy, a Dog, and a Frog.* New York, NY: Dial Press.

[B7] Mayer M. (1969). *Frog, Where Are You?* New York, NY: Dial Press.

[B8] Mayer M. (1971). *A Boy, a Dog, a Frog, and a Friend.* New York, NY: Dial Press.

[B9] Mayer M. (1973). *Frog on His Own.* New York, NY: Dial Press.

[B10] Mayer M. (1974). *Frog Goes to Dinner.* New York, NY: Dial Press.

[B11] Mayer M. (1975). *One Frog Too Many.* New York, NY: Dial Press.

[B12] McNerney M. W. Goodwin K. A. Radvansky G. A. (2011). A novel study: a situation model analysis of reading times. *Discourse Process* 48 453–474. 10.1080/0163853X.2011.582348

[B13] Newtson D. (1973). Attribution and the unit of perception of ongoing behavior. *J. Pers. Soc. Psychol.* 28 28–38. 10.1037/h0035584

[B14] Newtson D. Engquist G. (1976). The perceptual organization of ongoing behavior. *J. Exp. Soc. Psychol.* 12 436–450. 10.1016/0022-1031(76)90076-7

[B15] Newtson D. Engquist G. Bois J. (1977). The objective basis of behavior units. *J. Pers. Soc. Psychol.* 35 847–862. 10.1037/0022-3514.35.12.847

[B16] Radvansky G. A. (2012). Across the event horizon. *Curr. Dir. Psychol. Sci.* 21 269–272. 10.1177/0963721412451274

[B17] Radvansky G. A. Copeland D. E. (2010). Reading times and the detection of event shift processing. *J. Exp. Psychol. Learn. Mem. Cogn.* 36 210–216. 10.1037/a0017258 20053056

[B18] Radvansky G. A. D’Mello S. Abbott R. G. Morgan B. Fike K. Tamplin A. K. (2015). The fluid events model: predicting continuous task action change. *Q. J. Exp. Psychol.* 68 2051–2072. 10.1080/17470218.2015.1004354 25607590

[B19] Radvansky G. A. Zacks J. M. (2011). Event perception. *Wiley Interdiscip. Rev. Cogn. Sci.* 2 608–620. 23082236 10.1002/wcs.133 PMC3472805

[B20] Radvansky G. A. Zacks J. M. (2014). *Event Cognition.* Oxford: Oxford University Press.

[B21] Radvansky G. A. Zwaan R. A. Curiel J. M. Copeland D. E. (2001). Situation models and aging. *Psychol. Aging* 16 145–160. 10.1037/0882-7974.16.1.145 11302363

[B22] Reder L. M. Schunn C. D. (1999). “Bringing together the psychometric and strategy worlds: predicting adaptivity in a dynamic task,” in *Cognitive Regulation of Performance: Interaction of Theory and Application. Attention and Performance XVII*, eds Gopher D. Koriat A. (Cambridge, MA: MIT Press), 315–342.

[B23] Rinck M. Weber U. (2003). Who when where: an experimental test of the event-indexing model. *Mem. Cogn.* 31 1284–1292. 10.3758/bf03195811 15058689

[B24] Speer N. K. Zacks J. M. Reynolds J. R. (2007). Human brain activity time-locked to narrative event boundaries. *Psychol. Sci.* 18 449–455. 10.1111/j.1467-9280.2007.01920.x 17576286

[B25] Stevens S. S. Galanter E. H. (1957). Ratio scales and category scales for a dozen perceptual continua. *J. Exp. Psychol.* 54 377–411. 10.1037/h0043680 13491766

[B26] Stine-Morrow E. A. Loveless M. K. Soederberg L. M. (1996). Resource allocation in on-line reading by younger and older adults. *Psychol. Aging* 11 475–486. 10.1037/0882-7974.11.3.475 8893316

[B27] Wixted J. T. Ebbesen E. B. (1991). On the form of forgetting. *Psychol. Sci.* 2 409–415. 10.1111/j.1467-9280.1991.tb00175.x

[B28] Zacks J. M. Speer N. K. Reynolds J. R. (2009). Segmentation in reading and film comprehension. *J. Exp. Psychol. Gen.* 138 307–327. 10.1037/a0015305 19397386 PMC8710938

[B29] Zacks J. M. Speer N. K. Swallow K. M. Braver T. S. Reynolds J. R. (2007). Event perception: a mind-brain perspective. *Psychol. Bull.* 133 273–293. 10.1037/0033-2909.133.2.273 17338600 PMC2852534

[B30] Zacks J. M. Swallow K. M. (2007). Event segmentation. *Curr. Dir. Psychol. Sci.* 16 80–84. 10.1111/j.1467-8721.2007.00480.x 22468032 PMC3314399

[B31] Zwaan R. A. Magliano J. P. Graesser A. C. (1995). Dimensions of situation model construction in narrative comprehension. *J. Exp. psychol. Learn. Mem. Cogn.* 21 386–397. 10.3758/s13421-011-0179-8

[B32] Zwaan R. A. Radvansky G. A. (1998). Situation models in language comprehension and memory. *Psychol. Bull.* 123 162–185. 10.1037/0033-2909.123.2.162 9522683

[B33] Zwaan R. A. Radvansky G. A. Hilliard A. E. Curiel J. M. (1998). Constructing multidimensional situation models during reading. *Sci. Stud. Read.* 2 199–220. 10.1207/s1532799xssr0203_2

